# A DNA aptamer reveals an allosteric site for inhibition in metallo-β-lactamases

**DOI:** 10.1371/journal.pone.0214440

**Published:** 2019-04-22

**Authors:** Nazmul H. Khan, Anthony A. Bui, Yang Xiao, R. Bryan Sutton, Robert W. Shaw, Benjamin J. Wylie, Michael P. Latham

**Affiliations:** 1 Department of Chemistry & Biochemistry, Texas Tech University, Lubbock, Texas, United States of America; 2 Department of Cell Physiology & Molecular Biophysics, Texas Tech University Health Sciences Center, Lubbock, Texas, United States of America; Wake Forest University, UNITED STATES

## Abstract

The hydrolysis of β-lactam antibiotics by β-lactamase enzymes is the most prominent antibiotic resistance mechanism for many pathogenic bacteria. Out of this broad class of enzymes, metallo-β-lactamases are of special clinical interest because of their broad substrate specificities. Several *in vitro* inhibitors for various metallo-β-lactamases have been reported with no clinical efficacy. Previously, we described a 10-nucleotide single stranded DNA aptamer (10-mer) that inhibits *Bacillus cereus* 5/B/6 metallo-β-lactamase very effectively. Here, we find that the aptamer shows uncompetitive inhibition of *Bacillus cereus* 5/B/6 metallo-β-lactamase during cefuroxime hydrolysis. To understand the mechanism of inhibition, we report a 2.5 Å resolution X-ray crystal structure and solution-state NMR analysis of the free enzyme. Chemical shift perturbations were observed in the HSQC spectra for several residues upon titrating with increasing concentrations of the 10-mer. In the X-ray crystal structure, these residues are distal to the active site, suggesting an allosteric mechanism for the aptamer inhibition of the enzyme. HADDOCK molecular docking simulations suggest that the 10-mer docks 26 Å from the active site. We then mutated the three lysine residues in the basic binding patch to glutamine and measured the catalytic activity and inhibition by the 10-mer. No significant inhibition of these mutants was observed by the 10-mer as compared to wild type. Interestingly, mutation of Lys50 (Lys78; according to standard MBL numbering system) resulted in reduced enzymatic activity relative to wild type in the absence of inhibitor, further highlighting an allosteric mechanism for inhibition.

## Introduction

β-lactam antibiotics are the most widely prescribed class of antimicrobial drugs because of their high effectiveness and relatively low cost [1]. Consequently, the evolution of β-lactam antibiotic resistance in pathogenic bacteria is a major threat to human health. The production of β-lactamase enzymes, which catalyze the hydrolysis of the endocyclic amide bond of the β-lactam ring, is the most common mechanism for resistance to these antibiotics [2]. Based on sequence identity, there are four classes of β-lactamases. Classes A, C, and D are serine β-lactamases, which have a serine residue in their active site. Class B enzymes are zinc dependent metallo-β-lactamases (MBLs), which require one or two Zn^2+^ in their active site for catalysis [3, 4]. The most studied, clinically important chromosomally encoded MBLs are native to *Bacillus cereus* and *Stenotrophomonas maltophilia* [5]. In recent years, many new and highly transmissible MBLs have been identified [6]. For example, since its discovery in a Swedish male patient of Indian origin in 2009, New Delhi metallo-β-lactamase 1 (NDM-1) has emerged as the most dangerous threat in the rise of multi-drug resistant bacteria strains. Encoded in a highly transposable genetic element, NDM-1 can hydrolyze almost all β-lactam antibiotics including carbapenems [7]. Additionally, more than 140 allotypes of imipenemase (IMP) and Verona integron-encoded metallo-β-lactamase (VIM) type MBLs have been reported worldwide [8, 9]. Both IMP- and VIM-type enzymes show broad substrate specificity with high affinity for cephalosporins and carbapenems [8]. VIM-type MBLs are also capable of hydrolyzing 6-α-methoxypenicilin, a β-lactam antibiotic thought to be stable against β-lactamases [8–11]. Thus, developing novel inhibitors for MBLs is essential for the continued use of β-lactam antibiotics. Though extensive studies have been performed, no therapeutic inhibitors have been identified. This search is complicated by not fully understanding the role of the catalytic Zn^2+^ ions in the catalytic mechanism and architectural similarities of active sites of MBLs with some mammalian enzymes [12, 13]. Despite these challenges, several promising *in vitro* inhibitors of MBL have been identified. In most cases, these inhibitors were found to inhibit the enzyme by interacting with the active site residues or by chelating the Zn^2+^ ions [14–18] which would have toxic side effects in humans. For example, the anticancer antibiotic mithramycin inhibits MBL from *Bacillus cereus* (BcII MBL) by altering the structure of the enzyme and potentially disrupting the active site Zn^2+^ ions [19]. Some inhibitors have also been reported that mediate inhibition of MBL without interacting with the active site Zn^2+^. For example, camelid nanobodies inhibit VIM-4 by interacting with residues away from the active site [20].

For systemic treatment, it is essential to develop directed and selective therapies. To this end, oligonucleotide aptamers show promise as both drugs and drug delivery systems [21, 22]. Aptamers can recognize a target molecule with high affinity and specificity [21]. Advantages of nucleic acid aptamers, compared to traditional antibodies or nanobodies, include their smaller size, structural flexibility, low production cost and time, and little or no immunogenicity or toxicity [21–23]. Nucleic acid aptamers are not however without drawbacks: for example, they are very susceptible to degradation by endogenous nucleases and do not readily cross the plasma membrane both of which limits their bioavailability [24]. Previously, we identified oligonucleotide aptamers that act as inhibitors of *Bacillus cereus* 5/B/6 metallo-β-lactamase (hereafter referred to as 5/B/6 MBL; Sequence ID: AAA22562.1). We used SELEX [25] to select single-stranded DNA (ssDNA), double-stranded DNA (dsDNA), and RNA aptamers from random oligonucleotide starting libraries. A truncated 10-residue DNA (10-mer) was the most potent oligonucleotide inhibitor identified from a random 61-mer ssDNA library [14]. Note, the 5/B/6 MBL, which we use in our biochemical and structural studies, is a different isolate from the commonly used BcII MBL (Sequence ID: EEL64219.1). 5/B/6 MBL, which was isolated by Davies et al [26], and BcII MBL, which was isolated by Sabath et al [27] with crystal structures reported by Carfi et al [28] and Fabiane et al [29], share 93% sequence identity, and as we will show below are structurally the same.

In this paper, we couple kinetic and structural studies of the free and inhibited *Bacillus cereus* 5/B/6 MBL to understand the structure-activity relationships that underlie the inhibition by the 10-mer DNA. We structurally characterized the enzyme using X-ray crystallography and solution state NMR. Our structural data, including chemical shift perturbations from an NMR titration experiment, were leveraged to constrain a model of the enzyme-inhibitor complex. This model was confirmed by mutating enzyme residues found to interact with the 10-mer. Our data suggests that the association of 10-mer with 5/B/6 MBL during cefuroxime hydrolysis allosterically modulates the enzyme, culminating in uncompetitive enzymatic inhibition. This result identifies a novel site for future 5/B/6 and BcII MBL inhibitor design.

## Materials and methods

### *Bacillus cereus* 5/B/6 metallo-β-lactamase expression and purification

For expression and purification of unlabeled enzyme, *E*. *coli* BL21 (DE3) codon plus competent cells (Stratagene) were transformed with pET29 plasmid containing the structural gene of *B*. *cereus* 5/B/6 MBL. Transformed cells were grown at 37 °C in LB medium supplemented with 0.1 mM ZnSO_4_ and 50 μg/ml kanamycin. Enzyme overexpression was induced with 1 mM IPTG added at attenuance at 600 nm of 1.0; the cells were further grown for 12 hours at 20 °C. Cells were harvested by centrifugation, washed, and resuspended in 20 mM MOPS, 1 mM ZnSO_4_, pH 7.0 buffer (20/1 buffer). Then the cells were lysed via four passes through a French pressure cell. Cell lysate was centrifuged using a Fiberlite F21-8x50y rotor (Thermo Scientific) at 20000 rpm (47360 g) for 1 hour at 4 °C. Solid (NH_4_)_2_SO_4_ was added to the supernatant to a final concentration of 200 mM. Insoluble materials were removed by ultracentrifugation using a Beckman Ti 90 rotor at 90000 rpm (694000 g) for 2 hours at 4 °C. Supernatant was loaded onto a 25 ml POROS XS strong cation exchange column (Applied Biosystems) previously equilibrated with 20/1 buffer. This column was washed with 20/1 buffer. After elution of unbound materials, the bound enzyme was eluted with a step gradient to 20 mM MOPS, 1 mM ZnSO_4_, 200 mM (NH_4_)_2_SO_4_, pH 7. Fractions that contained the enzyme were loaded onto a 7×15.6 cm Bio-gel P-60 column previously equilibrated with 20/1 buffer. The column was washed with 20/1 buffer. Enzyme containing fractions were concentrated by using an Amicon concentrator (Cole-Parmer, USA) equipped with an YM-10 membrane (10000 molecular weight cutoff). After every step of the purification, enzyme activity was calculated with a modified method described by Kim *et al*. [14] and Davies *et al*. [30] and total protein was determined by the method of Lowry *et al*. [31] using bovine serum albumin for the standard. Enzyme purity was ascertained by specific activity and SDS-PAGE and was judged to be >98% pure. Glycerol (20% v/v final concentration) was added to the pure enzyme, which was stored at -20 °C. For NMR experiments, isotopically labeled enzyme was purified in the same way, however, the cells were grown in 2x M9 minimal medium (13.6 g/L Na_2_HPO_4_, 6.0 g/L KH_2_PO_4_, 1.0 g/L NaCl, 0.5 g/L Mg_2_SO_4_∙7H_2_O, pH 7.1) [32] supplemented with ^12^C- or ^13^C-d-glucose (3 g/L) and (^15^NH_4_)_2_SO_4_ (1 g/L) as the sole carbon and nitrogen sources, respectively, along with vitamins (1 μg/mL each biotin and thiamine) and kanamycin (50 μg/ml). Generally, ~40 and ~25 milligrams of purified enzyme were produced from per liter LB or minimal media, respectively.

### Selection of the 10-mer DNA

The process of the 10-mer selection by SELEX was described in Kim *et al*. [14]. The 10-mer (5’-CCAAACTTGG-3’) was purchased from Oligo Factory (Holliston, MA, USA) and used in all inhibition assays and NMR experiments without further purification.

### Enzyme kinetic studies

Using cefuroxime as the substrate, we measured 5/B/6 MBL activity in 50 mM MOPS, 1 mM ZnSO_4_, pH 7 through a spectrophotometric method described by Kim *et al*. [14]. In this protocol, the decrease in substrate absorbance (Δε_276nm_ = 4.42 mM^-1^ cm^-1^) was continuously monitored at 276 nm during hydrolysis using a Shimadzu UV160U spectrophotometer and a quartz cuvette with 0.1 cm pathlength. All assays were performed with a final concentration of 0.3 μM enzyme in a reaction volume of 300 μL. One unit of enzyme activity was defined as the amount of enzyme required for catalyzing the hydrolysis of 1 μmol of substrate in 1 minute at 30 °C. For inhibition assays, diluted enzyme was pre-incubated with or without 10-mer DNA aptamer in the buffer for 15 min at 25 °C. All kinetic measurements were repeated in at least triplicate. A global nonlinear curve fitting was performed to determine the inhibition type using the equation $v=\frac{V_{\text{max}\left(app\right)}*[S]}{K_{m\left(app\right)}+[S]}$ in GraphPad Prism 6.0 [33], where *v* is the reaction rate, $K_{m(app)}=\frac{K_{m}}{\alpha^{'}}$; *K*_*m*_ is the Michaelis-Menten constant, $V_{max(app)}=\frac{V_{max}}{\alpha^{'}}$; *V*_*max*_ is the maximum velocity and *V*_*max*_ = *k*_*cat*_ [*E*] (enzyme concentration), [*S*] substrate concentration and $\alpha^{'}=1+[I]/K_{I}^{'}$ (where [*I*] is the inhibitor concentration and $K_{I}^{'}$ is the inhibition constant). In the global fit, the *V*_*max*_, *K*_*m*_, and $K_{I}^{'}$ are shared in all datasets. IC_50_s (concentration of an inhibitor that inhibits 50% of the enzyme) were determined by plotting log of remaining activity as a function of inhibitor concentration.

### Crystallography

Crystallization trials for 5/B/6 MBL were carried out using the structure screen 1 + 2 HT-96 (Molecular Dimensions). The concentration of the enzyme sample used for the screening was 20 mg/ml in 5 mM MOPS and 0.5 mM ZnSO_4_ at pH 7.0. Crystals typically appear within a week when incubated at 23 °C. Further optimization was performed using 24-well VDX plates and 1 mL reservoir volume containing 0.1 M Tris at pH 8.5 and 8% w/v PEG 8000. Hanging droplets were comprised of 1 μL of reservoir solution and 1 μL of protein. Final X-ray data were collected at 100 K and a wavelength of 1.283 Å using beam line 7–1 at Stanford Linear Accelerator Center (SLAC). Data were integrated using XDS [34]. Merging and scaling were performed using AIMLESS [35]. Molecular replacement, starting from PDB ID 1BC2 [Fabiane et al 1998], and refinement were done with PHENIX [36]. COOT [37] was used for manual density fitting. The coordinates and structure factors have been deposited in the Protein Data Bank (http://pdb.org) under the PDB ID 6DJA. Table 1 summarizes the data collection and refinement statistics. Ramachandran statistics showed that 97.3% and 2.7% of all residues were in favored and allowed regions, respectively.

**Table 1:** Data collection and refinement statistics (molecular replacement).

	*Bacillus cereus 5/B/6* metallo-β-lactamase^†^
**Data collection**	
Space group	P3_2_21
Cell dimensions	
*a*, *b*, *c* (Å)	71.09, 71.09, 96.18
*a*, *β*, *γ* (°)	90, 90, 120
Resolution (Å)	61.56–2.48 (2.54–2.48) *
*R*_merge_	0.068 (0.337)
*I* / *σI*	43.3 (10.8)
Completeness (%)	99.5 (93.4)
Redundancy	31.7 (24.0)
**Refinement**	
Resolution (Å)	37.90–2.48
No. reflections	9331
*R*_work_ / *R*_free_	16.6% / 21.15%
No. atoms	1799
Protein	1728
Zn ion	2
Water	69
*B*-factors	24.92
Protein	24.23
_1_Zn^2+^/_2_Zn^2+^	46.71/87.13
Water	41.02
R.m.s. deviations	
Bond lengths (Å)	1.20
Bond angles (°)	0.008
Ramachandran statistics	
Favored regions	97.27%
Allowed regions	2.73%
Disallowed regions	0.00%

^†^One crystal was used for structure determination.

*Values in parentheses are for highest-resolution shell.

### NMR spectroscopy and sequential backbone assignments

NMR experiments were performed on an Agilent DD2 600 MHz spectrometer (Santa Clara, CA, USA) equipped with a z-axis pulsed field gradient room temperature HCN probe. Standard 3D gradient-selected, sensitivity enhanced triple resonance backbone assignment experiments (HNCACB/CBCAcoNH, HNCA/HNcoCA and HNCO/HNcaCO) [38, 39] were recorded on an ~1.4 mM enzyme sample in 20 mM MOPS, 1 mM ZnSO_4_, pH 7 at a set point of 25 °C. Spectra were processed and analyzed with the NMRPipe/NMRDraw software package [40] and CCPN analysis program [41]. The secondary structure of 5/B/6 MBL was predicted by the program TALOS+ [42], using the ^1^HN, ^15^N, ^13^CO, ^13^C^α^, and ^13^C^**β**^ chemical shift resonance assignments, and compared with the X-ray crystal structure using PyMol [43]. Additionally, the ^1^HN, ^15^N, ^13^CO, ^13^C^α^, and ^13^C^**β**^ chemical shift resonance assignments were submitted to the CS-ROSETTA [44] server (https://csrosetta.bmrb.wisc.edu/csrosetta/submit) and 3000 structures were calculated using default parameters. Note, this structure was only used for diagnostic purposes, to confirm that the measured NMR chemical shifts conform to our X-ray crystal structure. Backbone chemical shift assignments have been deposited in the Biological Magnetic Resonance Data Bank (http://www.bmrb.wisc.edu/) under BMRB ID 27564.

### NMR titration experiments

NMR titration experiments were performed by combining 750 μM uniformly ^15^N-labeled enzyme with 0, 0.125, 0.25, 0.375, 0.50, 0.625, 0.75, 0.875, 1.0, 1.25, 1.50, 2.0, and 4.0 molar equivalents of the 10-mer aptamer. Gradient-selected, sensitivity enhanced ^1^H-^15^N HSQC [45, 46] spectra were recorded on an Agilent DD2 600 MHz spectrometer at a set temperature of 25 °C and were processed and analyzed with NMRPipe and CCPN analysis. Chemical shift perturbations at a given inhibitor concentration (*δ*_*NH*,*i*_) were calculated according to $\delta_{NH,i}=\sqrt{{\left(\delta_{H,apo}-\delta_{H,i}\right)}^{2}+{0.1*\;(\delta_{N,apo}-\delta_{N,i})}^{2}}$; where *δ*_*H*_ and *δ*_*N*_ are the ^1^HN and ^15^N chemical shifts in the free state (*apo*) and bound to inhibitor (*i*), respectively. Binding affinities were calculated by fitting the chemical shift changes of the enzyme as a function of inhibitor concentration to $y=A[(B+x)-\sqrt{\left\{{\left(B+x\right)}^{2}-4x\right\}}]$; where *A* = (maximum chemical shift change)/2, *B* = 1+K_D_/[protein], *y* = chemical shift change, and *x* = [ligand]/[protein].

### 10-mer-5/B/6 MBL docking

Models of the 10-mer-5/B/6 MBL complex were calculated from molecular docking simulations using the HADDOCK [47] software package (http://milou.science.uu.nl/services/HADDOCK2.2/haddockserver-easy.html). Our crystal structure of 5/B/6 MBL and a model of the 3-D 10-mer hairpin structure, which was calculated in 3D-NuS [48] (https://iith.ac.in/3dnus/index.html), were used as starting structures. Docking restraints were derived from the chemical shift perturbations observed during the 10-mer NMR titration. “Active” residues, which are those experimentally identified to take place in the binding reaction, in the docking simulations included Thr76, Lys78, Phe103, Lys104, Lys107, and Tyr208 of 5/B/6 MBL, whereas all nucleotides in the 10-mer were considered “active.” The residue numbering is presented according to the standard MBL numbering system [49, 50]. “Passive” residues, which are solvent exposed neighbors to “active” residues, were defined automatically by HADDOCK. The 200 calculated structures were automatically classified into 8 clusters.

### Site-directed mutagenesis

Site-directed lysine to glutamine mutations were generated through a modified QuikChange (Stratagene) protocol. Plasmids encoding these mutants were transformed into *E*. *coli* BL21 (DE3) codon plus cells for over-expression. Protein purification and activity and inhibition assays were performed as described above.

## Results

### Enzyme kinetic studies suggest a novel site for aptamer binding

**Fig 1:**
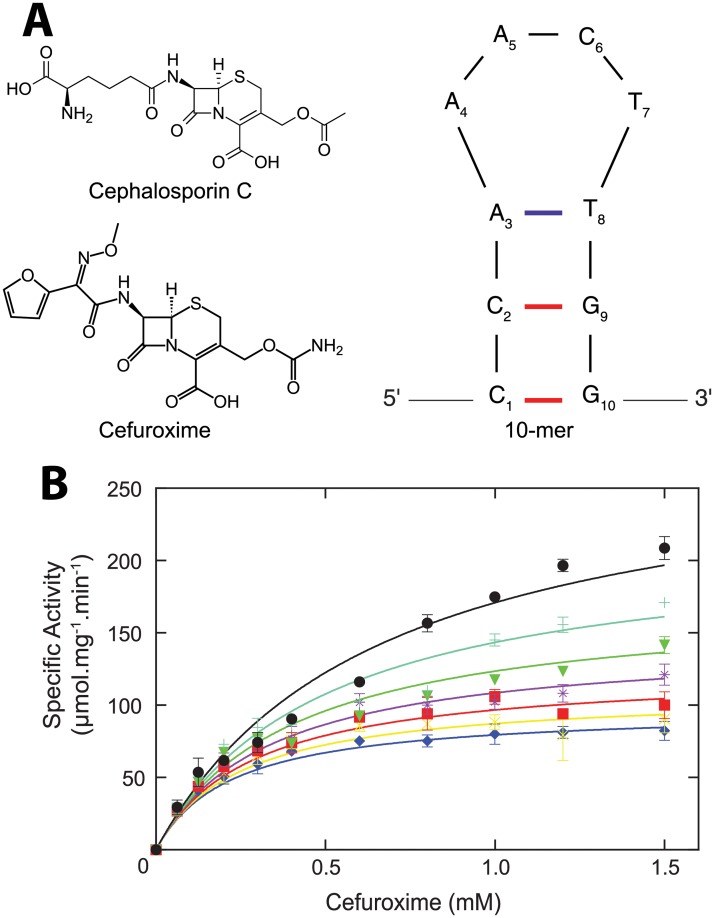
Inhibition of 5/B/6 MBL by the 10-mer aptamer during cefuroxime hydrolysis. A) Chemical structures of the β-lactam antibiotics cephalosporin C and cefuroxime. Also shown is the M-fold predicted secondary structure of the 10-mer DNA aptamer. B) Plot of the specific activity versus cefuroxime concentration. Points and error bars are the average and standard deviation of at least three measurements. Solid lines represent the non-linear regression calculation (global correlation coefficient R^2^ = 0.964), which shows the uncompetitive inhibition pattern during cefuroxime hydrolysis. Black circle: 10-mer concentration [I] = 0 (local correlation coefficient R^2^ = 0.969); cyan: [I] = 20 nM (R^2^ = 0.957); green: [I] = 40 nM (R^2^ = 0.961); magenta: [I] = 60 nM (R^2^ = 0.965); red: [I] = 80 nM (R^2^ = 0.949); yellow: [I] = 100 nM (R^2^ = 0.913); and blue: [I] = 120 nM (R^2^ = 0.960).

To understand the mechanism of 10-mer inhibition of 5/B/6 MBL, we quantified Michaelis-Menten enzyme kinetics by monitoring the hydrolysis of the antibiotic cefuroxime (second generation semisynthetic cephalosporin with a furan containing side chain, Fig 1A). For wild type, uninhibited 5/B/6 MBL, *K*_*m*_, *V*_*max*_, and *k*_*cat*_ were calculated to be 1.1 ± 0.1 mM, 370 ± 20 μmol∙min^-1^∙mg^-1^, and 164 ± 8, respectively, from a non-linear regression plot (black points in Fig 1B). The 10-mer aptamer inhibits 5/B/6 MBL cefuroxime hydrolysis with an IC_50_ of 120 ± 5 nM (S1A Fig). In contrast to the noncompetitive inhibition of 5/B/6 MBL by the 10-mer during cephalosporin C (a cephalosporin antibiotic with a diamino adipoyl side chain, for which a *K*_*m*_ and *V*_*max*_ of 0.39 mM and 1333 μmol∙min^-1^∙mg^-1^ for the 5/B/6 MBL, respectively [51], Fig 1A) hydrolysis [14], uncompetitive inhibition (Fig 1B and S1B Fig) was observed for the hydrolysis of cefuroxime with a calculated inhibition constant ($K_{I}^{'}$) of 63 ± 3 nM. Thus, the inhibition pattern and kinetic parameters of 5/B/6 MBL differ between substrates and suggests that the 10-mer does not bind 5/B/6 MBL competitively. Note, the 10-mer was originally derived from a 30 nucleotide DNA obtained from our SELEX experiment. As part of that experiment, we showed that the inhibitory effect of this DNA aptamer resulted solely from the 10-mer sequence. Deleting the flanking DNA sequences upstream and downstream of the predicted 10-mer hairpin structure resulted in the same inhibition of 5/B/6 MBL activity as the 30-mer DNA, and the isolated flanking sequences did not inhibit on their own [14].

Uncompetitive inhibitors require the formation of the enzyme-substrate complex in order to interact with the enzyme and are characterized by decreases in both *K*_*m*_ and *V*_*max*_, as seen here for cefuroxime in Fig 1B and S1B Fig. During uncompetitive inhibition, an enzyme-catalyzed reaction becomes blocked beyond ES formation [52]. On the other hand, noncompetitive inhibitors can either bind to the enzyme alone or to the enzyme-substrate complex and are characterized by an increase in *K*_*m*_ and decrease in *V*_*max*_. Therefore, the 10-mer aptamer inhibition pattern exhibited for each substrate indicates that the interaction of the inhibitor with 5/B/6 MBL probably occurs at an allosteric site distal to the active site (i.e. is not competitive with the substrate) [20, 53].

### Structure of wild type *B*. *cereus* 5/B/6 metallo-β-lactamase

**Fig 2:**
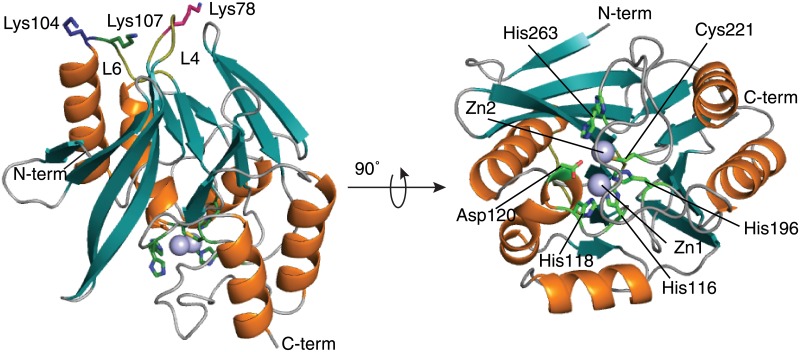
X-ray crystal structure of 5/B/6 MBL shows the typical MBL fold. Secondary structural elements are colored deep teal for beta sheets and orange for alpha helices; whereas, turns/coils are colored grey and the Zn^2+^ atoms are represented by light blue spheres. The left panel shows the overall enzyme structure with lysine residues at the allosteric site in Loop 4 (L4) and Loop 6 (L6). The right structure shows the active site residues that are responsible for coordinating the two Zn^2+^ ions.

To put the mechanism of inhibition into a structural context, X-ray crystallography trials were initiated for the aptamer-free and 10-mer-bound enzyme. The 2.5 Ångström (Å) resolution crystal structure of aptamer-free 5/B/6 MBL with both Zn^2+^ ions bound is shown in Fig 2. Crystal structures of several B1 metallo-β-lactamases have been determined and can be found on the MBLED (Metallo-Beta-Lactamase Engineering Database; http://www.mbled.uni-stuttgart.de) [54]. Subclass B1 MBLs display a αβ/βα sandwich fold in their tertiary structures with a consensus sequence of HXHXD(X)_i_H(X)_j_C(X)_k_H (in single letter code for amino acids where X = any amino acid, i = 55–74, j = 18–24, and k = 37–41), with H, D, and C residues coordinating the Zn^2+^ ions [15]. Our structure shows a similar fold. Table 1 contains the crystallography data collection and refinement statistics.

**Fig 3:**
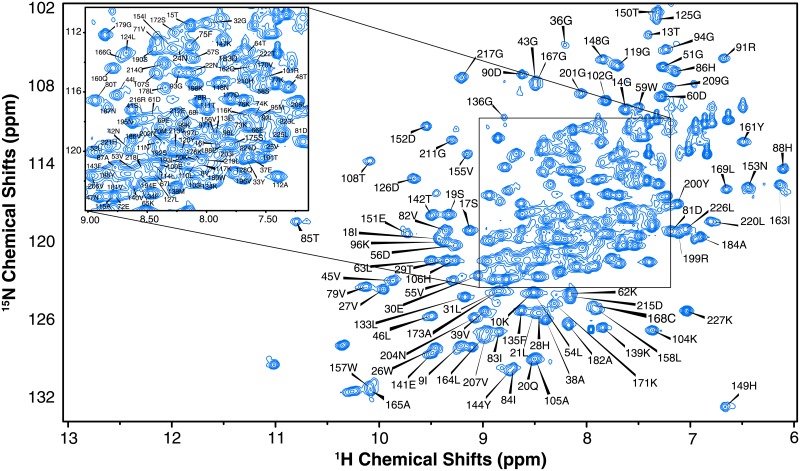
2D ^1^H-^15^N HSQC spectra of 5/B/6 MBL. Amino acid assignments are indicated. Inset is a magnification of the more crowded region.

Because of difficulties in obtaining diffraction quality crystals of the 10-mer-bound 5/B/6 MBL, solution state NMR studies were also initiated. The ^1^H-^15^N HSQC of aptamer-free 5/B/6 MBL is shown in Fig 3; over 87% (199 non-proline residues out of 228 total residues) of the backbone ^1^HN, ^15^N, ^13^CO, ^13^C^α^, and ^13^C^**β**^ resonances were assigned. Main chain assignments were not obtained for Glu28-Lys34, Glu39, Lys50, Ser62, Pro68, Ser69, Ala140, Glu168, Glu169, Pro170, Ser176-Asn179, Asn184, Pro192, Gly193, Pro206, Ser227, Asn233, and Pro261. Most of the unassigned residues are found either in turns or loop regions. The secondary structure propensities were calculated from the NMR data by TALOS+ and compared with those of the X-ray crystal structure. The length and position of the secondary structure elements agreed between the X-ray crystallography and solution state NMR data sets (S2 Fig). The CS-ROSETTA calculated solution NMR structure also shows the typical αβ/βα sandwich fold for metallo-β-lactamases (S3A Fig). Because many of the loop resonances were not assigned and the Zn^2+^ ions were not present in the CS-ROSETTA modeling, small differences exist between the X-ray crystal and solution NMR structures. This is shown in S3B Fig where the α-helix and β-sheet regions of the X-ray crystal and NMR structures were aligned in PyMOL to compare the two structures. The C^α^ r.m.s.d. for regions of secondary structure is 2.37 Å indicating that the two structures are indeed similar. Together, these structural data are the starting point for understanding the structure of the 10-mer aptamer-inhibited 5/B/6 MBL.

### NMR titration experiments confirm that the 10-mer does not bind to the enzyme active site

**Fig 4:**
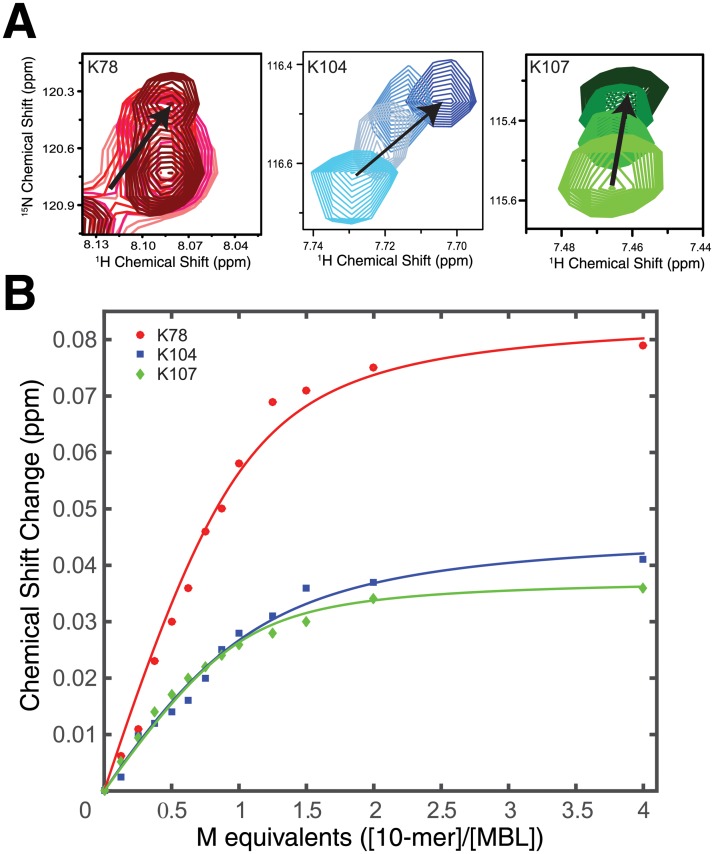
NMR titration of 5/B/6 MBL with the 10-mer aptamer reveals the site of aptamer binding. A) 2D ^1^H-^15^N HSQC spectra of lysine residues that showed chemical shift perturbation upon the addition of 10-mer. The direction of the movement for each peak is shown with a black arrow. The lightest color represents the no inhibitor condition and the darkest color represents presence of 3 mM of the 10-mer (i.e., 4.0 molar equivalents). B) Binding curves for the three lysine residues. The calculated K_D_s from individual fits of the Lys78, Lys104, and Lys107 chemical shift change are 125 ± 4 nM, 233 ± 6 nM, and 100 ± 3 nM, respectively. Errors in the K_D_ are from the fit.

We next performed NMR titration experiments to determine the location of 10-mer binding and the binding affinity (S4 Fig). Changes in the chemical environment of NMR active nuclei upon ligand binding are easily detected by monitoring chemical shift perturbations (CSPs) [55]. While the vast majority of residues experience no change in resonance position upon addition of 10-mer (S4 Fig), six residues showed significant CSPs upon titrating with increasing concentrations of 10-mer (0 to 4 molar equivalents): Thr76, Lys78, Phe103, Lys104, Lys107, and Tyr208. Interestingly, all of these residues are on the opposite side of the enzyme from the active site in three structurally adjacent loops (L4, L6, L13). Among these residues were three lysines (78, 104, and 107) on loops L4 and L6, shown in the crystal structure in Fig 2A, that make up a basic patch. The side chain amines of Lys78 and Lys107 are 11.7 Å away from each other providing ample space to occupy the loop region of the 10-mer (~8.7 Å distance between A5 and C6 of the aptamer). Moreover, the distance between the backbone amide groups of Lys104 and Tyr208 is ~22 Å, which is within the range of the length of a 10-mer hairpin structure (~20 Å). A spectral overlay highlighting the chemical shift perturbations between the reference and several inhibitor concentrations for these lysine residues is shown in Fig 4A. Since binding of 10-mer to enzyme occurred in the fast exchange regime of the chemical shift timescale, binding affinities were extracted from fits of the CSPs (Fig 4). While the CSPs are small in magnitude, likely reflecting that the interaction between 10-mer and 5/B/6 MBL occurs largely through side chain interactions, we were able to analyze the data to determine binding affinities. Typical hyperbolic binding curves were observed for all three lysine residues, shown in Fig 4B, and K_D_s of 100–233 nM were obtained from fits to a two-state quadratic binding isotherm. Note that this binding affinity is comparable to the $K_{I}^{'}$ obtained from analysis of the kinetic inhibition data (63 ± 3 nM).

It was previously hypothesized that the 10-mer exerts its inhibition by binding near the active site thereby perturbing the coordination of Zn^2+^ ions [14]. Significantly, no CSPs were observed in the HSQC spectra for the Zn^2+^-coordinating active site residues (His116, His118, Asp120, His196, Cys221 and His263; S4 Fig) indicating that the 10-mer does not strip the catalytic ions. This binding of aptamer to a site away from the active site supports the hypothesis of allosteric inhibition of the 5/B/6 MBL by the 10-mer.

### Molecular docking identifies probable interaction of the 10-mer with 5/B/6 MBL at the allosteric site

**Fig 5:**
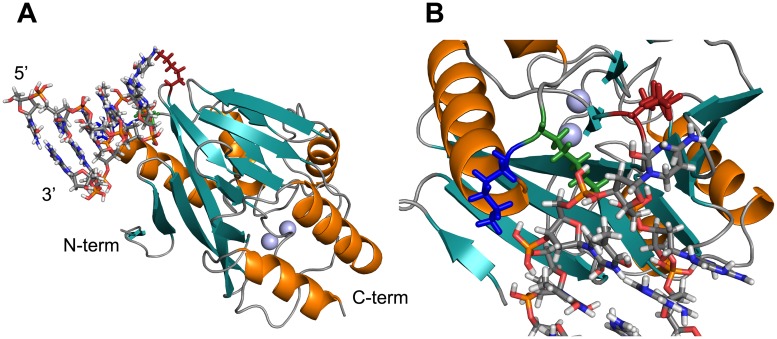
Model of the 10-mer bound to 5/B/6 MBL provides a picture of the inhibited state. Model of the 10-mer bound to 5/B/6 MBL. The lowest energy HADDOCK structure is given, while a representative structure from the seven other clusters is given in S5 Fig. The coloring of secondary structural elements for the 5/B/6 MBL follows Fig 2, and the red, blue and green sticks denote K78, K104, and K107, respectively. The 10-mer is shown as salmon sticks and the two Zn^2+^ ions are light blue spheres. A) The overall structure of the enzyme-10-mer complex. B) A closer view of the enzyme-10-mer interaction site.

For a low-resolution visualization of the 10-mer–enzyme complex, we performed molecular docking simulations using HADDOCK [47]. 200 models of the complex were calculated using our X-ray crystallography model and a hairpin model of the 10-mer aptamer, which was calculated in 3D-NuS [48]. Constraints were placed between the six 5/B/6 MBL residues that experienced CSPs and all ten residues of the 10-mer. Fig 5 and S5 Fig show the resulting structures of the simulated enzyme-aptamer complexes with different orientations of the 10-mer.

The docking models suggest that loops L4 and L6 predominantly interact with the 10-mer (Fig 5A). In the majority of the representative models, Lys104 (5 of 8 models–S5a, S5b, S5c, S5e and S5g Fig) and Lys107 (7 of 8 –not seen in S5c Fig) form ionic interactions with the ribose-phosphate backbone of the 10-mer. Interestingly, there are four models where these basic residues recognize A-T or C-G base pairs in the model stem loop DNA (Fig 5 and S5c, S5d and S5f Fig). A similar story is also found for Lys78: it forms ionic interactions with the DNA backbone in 3 of 8 models (Fig 5 and S5d and S5g Fig), or in one interesting case (S5b Fig), Lys78 hydrogen bonds with the imino nitrogen of Cyt6, which is in the proposed loop of the 10-mer. In half of the models (S5a, S5b, S5d and S5e Fig), the hydroxyl group of Tyr208 on L13 forms a hydrogen bond with the backbone of the DNA. Lastly, we did not observe any direct interactions in the HADDOCK models between the 10-mer and Thr76 and Phe103, so the CSPs for these residues must be due to indirect effects from aptamer binding. Note, that the small backbone amide CSPs observed in Fig 5 and S5 Fig are indeed consistent with these models, which suggest that the binding of 10-mer to 5/B/6 MBL is mediated through side chain interactions. Thus, we hypothesize that the 10-mer interacts with the lysine residues (Fig 5B) on L4 and L6 and disrupts a network of residues towards the Zn2 ion.

### Mutations confirm the presence of an allosteric site on B. cereus 5/B/6 metallo-β-lactamase

**Fig 6:**
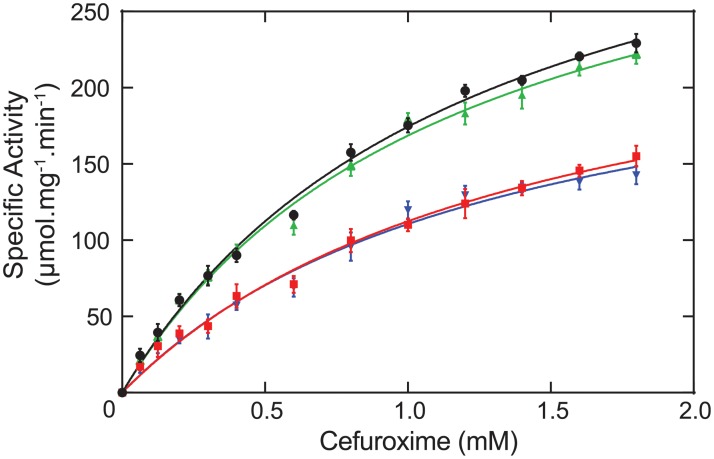
Steady-state kinetics of cefuroxime hydrolysis by different mutants of 5/B/6 MBL confirms 10-mer binding site. Activities of three mutant enzymes were compared with the activity of wild type enzyme, where black is the wild type enzyme, and green, blue, and red correspond to K104Q/K107Q, K78Q, and K78Q/K104Q/K107Q, respectively. The solid lines represent fits to the Michaelis-Menten equation with the resulting kinetic values reported in Table 2. Correlation coefficients (R^2^) of 0.993, 0.981, 0.990, and 0.978 where obtained for wild type, K78Q, K104Q/K107Q, and K78Q/K104Q/K107Q, respectively. Error bars indicate the standard deviation of five replicates.

To corroborate our binding model, three lysine-to-glutamine mutants were made via site-directed mutagenesis (K78Q, double mutation K104Q/K107Q, and triple mutation K78Q/K104Q/K107Q). As these mutations are far from the active site, it was expected that there would be no change in enzymatic activity during cefuroxime hydrolysis. Specific activities of wild type and mutant enzymes are shown in Table 2. Without the inhibitor, the double mutant (K104Q/K107Q) showed no significant change in activity as compared to wild type. On the other hand, the single mutant (K78Q) and triple mutant (K78Q/K104Q/K107Q) enzymes showed ~30% less activity than wild type enzyme. Analysis of the steady-state kinetics data (Fig 6) indicates that there are statistically significant (p-value = 0.0018) decreases in *k*_*cat*_ for K78Q and K78Q/K104Q/K107Q and no change in *K*_*m*_ (Table 2) as compared to wild type enzyme. This decrease in *k*_*cat*_ would likely have minimal effect on the activity of mutated enzyme *in vivo*; nevertheless, this result suggests that an allosteric network extends through the interior of the enzyme from the active site. Finally, inhibition of the three mutant enzymes by 10-mer was also studied. As shown in Table 2, no significant inhibition was observed in any of the mutant enzymes as compared to wild type, confirming our NMR and HADDOCK results that the aptamer must bind to these residues. Thus, disruption of ionic interactions between these lysine residues and the DNA leads to an inability of the 10-mer to bind the enzyme. This is consistent with our structural models where the inhibition by the aptamer occurs through binding to a unique allosteric binding site (Fig 5).

**Table 2:** Steady-state parameters of 5/B/6 MBL mutants.

Enzyme	Cefuroxime Hydrolysis					
	No 10-mer^a^			+ 500 μM 10-mer^a^		
	K_m_ (mM)	k_cat_ (s^-1^)	k_cat_/K_m_ (mM^-1^·s^-1^)	K_m_ (mM)	k_cat_ (s^-1^)	k_cat_/K_m_ (mM^-1^·s^-1^)
Wild type	1.2 ± 0.1	164 ± 8	133± 5	0.2 ± 0.1	36 ± 2	176 ± 30
K78Q	1.5 ± 0.1	117 ± 16	80 ± 7	1.2 ± 0.1	104± 6	89 ± 5
K104Q/K107Q	1.2 ± 0.1	154 ± 6	131 ± 7	1.2 ± 0.1	157 ± 11	126 ± 9
K78Q/K104Q/K107Q	1.3 ± 0.1	109 ± 10	82 ± 5	1.2 ± 0.1	109 ± 8	89 ± 9

^a^Kinetic values and associated errors represent the average of at least five replicates and their standard deviation.

## Discussion

Here, we described the structural basis for nanomolar uncompetitive inhibition of *B*. *cereus* 5/B/6 metallo-β-lactamase during cefuroxime hydrolysis by a 10-nucleotide DNA aptamer. We observe that the inhibition pattern of the 10-mer differs substrate-to-substrate (noncompetitive for cephalosporin C [14], uncompetitive for cefuroxime); thus, the kinetic mechanism details for the inhibition by the 10-mer are different for the two substrates. Different reaction mechanisms have been proposed for NDM-1 MBL hydrolysis of carbapenem and cephalosporin β-lactams [56, 57]. Like FEZ-1 MBL, the activity of the 5/B/6 MBL depends in part on the chemical moiety at the C-7 position of cephalosporin substrates, as the presence of an α-methoxy group at C-7 of cefoxitin has a negative effect on 5/B/6 MBL activity when compared to cephalothin hydrolysis [58]. Other differences could come from the charged groups in cephalosporins, which could interact electrostatically with the enzyme [59]. The difference in kinetic mechanism could then be extended to the inhibition pattern of the 10-mer on cephalosporin antibiotic hydrolysis by 5/B/6 MBL.

Our 2.5 Å crystal structure of aptamer-free 5/B/6 MBL overlays favorably with the structure of the closely related BcII enzyme (~93% sequence identity; PDB ID: 1BC2; C^α^ r.m.s.d. = 1.08 Å). Additionally, our structure also overlays extremely well with the crystal structures of NDM-1, VIM-2, and IMP-1 MBLs (PDB IDs: 3RKJ, 4NQ2, and 1DDK) with C^α^ r.m.s.d.’s of 1.07, 0.71, and 0.98 Å, respectively, highlighting the highly conserved nature of these folds. Interestingly, our structure shows that in 5/B/6 MBL Zn2 has a higher B-factor compared to Zn1, indicating that Zn2 exhibits a partial occupancy in the active site pocket unlike Zn1. Coupled to this, the active site residue His263 is found to be in two conformations in the X-ray structure (S6 Fig). This result suggests that His263 plays an important role in directing Zn2 to its location within the active site by acting as a gate locking Zn2 in place once it is bound. In fact, molecular dynamics simulations, directed evolution, and NMR relaxation experiments have suggested that the presence of a glycine residue at position 262, which is present in 5/B/6 MBL, increases the flexibility of His263 and other active site residues, alters the coordination of the Zn^2+^ ions, and affects the substrate specificity of the MBL [60–62].

Like antibody-antigen binding reactions, aptamers interact with their target through structural recognition [63, 64]. Usually, unpaired nucleotides in loop or bulge regions of a ssDNA molecule are involved in recognition of the protein through hydrogen bonding, which imparts the specificity of the aptamer-protein interaction [65]. Therefore, we hypothesize that the 10-mer in this study also interacts with 5/B/6 MBL through the loop region of its hairpin structure. Moreover, charged amino acids are generally found on the surface of proteins, and play many essential roles including the formation of protein-nucleic acid complexes [66]. To compensate for the negatively charged phosphate backbone, the protein-nucleic acid binding regions are primarily positively charged. For 5/B/6 MBL, the change in chemical shifts (and therefore the chemical environment) of the three positively charged lysine residues distal from the active site are indicative of 10-mer interaction at that site (Fig 4A). The shortest distance between the active site and inhibitor binding site is 25 Å, as measured from His116 to Lys78 –too far away for the aptamer to interact directly with both sites. However, any allosteric changes to the active site must be subtle, as the amide NMR chemical shifts of active site residues are not perturbed by 10-mer binding. One possibility is that the 10-mer binding could alter conformational changes or local dynamics around the active site required for catalysis [57, 67, 68]. Our proposed structural models for the enzyme-inhibitor complex (Fig 5 and S5 Fig) lack experimental restraints on the 10-mer structure in solution. Clearly, an experimentally derived structure of the 10-mer and additional restraints between aptamer and 5/B/6 MBL are required to fully understand the nature of this interaction. Nevertheless, instead of a potential therapeutic, the 10-mer should be viewed as a tool–the low-resolution models of the complex provided by the NMR titration and HADDOCK and the mutagenesis data have allowed us to define a novel allosteric site on this enzyme, which could now be subjected to rational drug design.

Interestingly, five of the six residues in 5/B/6 MBL that were perturbed upon the 10-mer binding and were used as restraints in the HADDOCK docking simulations are also conserved in the BcII MBL enzyme–Lys104 is a glutamine in the BcII MBL. This immediately suggests that the 10-mer will also bind to the BcII MBL, since these enzymes share ~93% sequence identity. In fact, preliminary studies have shown that the 10-mer does indeed inhibit cephalosporin antibiotic hydrolysis by the BcII MBL (R.W.S. personal communication). The K104Q/K107Q double mutation of 5/B/6 MBL, which contains the analogous BcII MBL Gln76 (103), was not inhibited by the 10-mer aptamer (Table 2). However, this could be due to the additive effect of losing two charge-charge interactions with the 10-mer; whereas, BcII MBL would retain one. A more in-depth mutagenesis study is necessary to tease these effects out, while the effect of the 10-mer on BcII MBL can be directly tested. Sequence alignment of the 340 subclass B1 sequences in the MBLED reveals that the basic residues utilized by the 10-mer for binding to 5/B/6 MBL are each present in ~30% of sequences. Thus, the 10-mer will probably not be a general inhibitor of subclass B1 MBLs. Nevertheless, given the high degree of structural similarity between the 5/B/6 MBL, NDM-1, VIM-4, and IMP enzymes, it is tempting to hypothesize that related allosteric sites also exist within these enzymes. In fact, epitope mapping of the VIM-4 inhibitory nanobody, which also utilizes an allosteric mechanism for inhibition, revealed that the nanobody binds VIM-4 MBL on the structurally analogous L6 that contains residues Lys104 and Lys107, which are perturbed in our NMR titration experiments, in 5/B/6 MBL [20]. Thus, there is very strong evidence that the allosteric site described herein is a common motif among several highly transmissible β-lactam neutralizing metallo-enzymes.

Allosteric drugs are used as therapeutics for several human diseases. The most common uses of allosteric modulators are for neurotransmitter receptor proteins in central nervous system disorders. For example, benzodiazepines act as positive modulators to enhance the effect of γ-amino butyric acid (GABA) by interacting with GABA_A_ receptors; whereas, 6,7-dihydropyrazolo[1,5-a]pyrazin-4(5H)-one acts as the negative modulator of metabotropic glutamate receptor-2 (mGluR2) [69]. To treat viral infection, use of allosteric inhibitors is also well established. For example, dasabuvir, an hepatitis C virus NS5B polymerase inhibitor, is used against chronic viral infection [70], and noncompetitive binding non-nucleotide reverse transcriptase inhibitors are a critical components of combination therapy used to treat HIV [71]. The development of similar allosteric inhibitors for bacterial enzymes has received attention very recently [20]. The alarming problem of antibiotic resistance has gained considerable interest and has pushed the discovery of new therapeutic agents to fight against bacterial infection. Because of the consensus active site architecture, finding effective inhibitors for MBLs have been mostly limited to orthosteric investigations. Due to their toxicity to the human body, divalent cation chelating agents specifically for MBLs are impossible to develop [14, 16, 72, 73]. Therefore, targeting allosteric sites for developing new drugs has great potential, as it is likely that specific allosteric modulators can be found for a unique site on the target protein that consequently avoids adverse effects to the human host [69, 74].

## Supporting information

S1 Fig Kinetic assays for *Bacillus cereus* 5/B/6 metallo-β-lactamase during cefuroxime hydrolysis. A) Determination of IC_50_ (120 ± 5 nM) of 10-mer for *Bacillus cereus* 5/B/6 metallo-β-lactamase during cefuroxime hydrolysis. B) Lineweaver-Burke double reciprocal plot of the data in Fig 1B highlighting the uncompetitive inhibition pattern.
(EPS)

S2 Fig Comparison of secondary structure elements derived from solution NMR data with those of the x-ray crystal structure of 5/B/6 MBL. Prediction of secondary structure elements from the NMR data was derived from backbone NMR chemical shifts using TALOS+ and are shown in orange bars for α-helices and cyan bars for β-sheets. Grey bars indicate unassigned residues. Secondary structure elements of 5/B/6 MBL x-ray crystal structure are indicated in orange rectangles for α-helices and cyan arrows for β-sheets. PDB ID: 6DJA was used to determine secondary structural elements from the crystal structure.
(EPS)

S3 Fig Solution NMR structure of 5/B/6 MBL. (A) The ^1^HN, ^15^N, ^13^CO, ^13^Cα, and ^13^Cβ chemical shift resonance assignments were submitted to the CS-ROSETTA and 3000 structures were calculated using default parameters. The lowest energy structure is shown here as a representative of all structures. The zinc ions coordinating the active site residues are not shown here. (B) Overlay of CS-ROSETTA derived model of 5/B/6 MBL (olive green) and the X-ray crystal structure (light green), which highlights the similarity of the secondary structure and the overall fold.
(EPS)

S4 Fig Overlay of 2D 1H-15N HSQC spectra for the NMR titration of 5/B/6 MBL with the 10-mer aptamer. Green, orange, red, light blue, and dark blue contours represent 0, 0.5, 1.0, 2.0, and 4 M equivalents of 10-mer DNA, respectively, titrated into 0.75 mM ^15^N-labeled 5/B/6 MBL. Data were collected at 600 MHz and 25 °C. Assignments are given for peaks in the active site (His86, His88, Asp90, His149, Cys168 and His210) as well as for peaks that titrated with 10-mer (denoted with arrows that highlight the direction of the chemical shift movement).
(EPS)

S5 Fig Structural models of the 10-mer-enzyme complex. These models were calculated through HADDOCK molecular docking. The coloring of secondary structural elements for the 5/B/6 MBL follows Fig 2, and the red, blue and green sticks denote Lys50, Lys76, and Lys77, respectively. The 10-mer is shown as salmon sticks and the two Zn2+ ions are light blue spheres.
(EPS)

S6 Fig The two conformational states of His210, an active site residue. These states arise due to partial occupancy of Zn2. This residue might play some important roles in directing Zn2 to its binding position, which may act as a gate to hold the Zn1.
(EPS)

## Abbreviations

5/B/6: *Bacillus cereus* 5/B/6
BcII: *Bacillus cereus* 569/H9
HADDOCK: Highly Ambiguous Data-driving DOCKing
HSQC: heteronuclear single quantum coherence
MBLs: metallo-β-lactamases
NDM-1: New Delhi metallo-β-lactamase 1
NMR: nuclear magnetic resonance
SLAC: Stanford Linear Accelerator Center
VIM-4: Verona integron-encoded metallo-β-lactamase

## References

[1] Perez F, Endimiani A, Hujer KM, Bonomo RA. The continuing challenge of ESBLs. Current Opinion in Pharmacology. 2007;7(5):459–69. 10.1016/j.coph.2007.08.003 17875405 PMC2235939

[2] Frere JM. Beta-lactamases and bacterial resistance to antibiotics. Molecular Microbiology. 1995;16(3):385–95. 7565100 10.1111/j.1365-2958.1995.tb02404.x

[3] Bebrone C. Metallo-β-lactamases (classification, activity, genetic organization, structure, zinc coordination) and their superfamily. Biochemical Pharmacology. 2007;74(12):1686–701. 10.1016/j.bcp.2007.05.021 17597585

[4] Palzkill T. Metallo-β-lactamase structure and function. Annals of the New York Academy of Sciences. 2013;1277:91–104. 10.1111/j.1749-6632.2012.06796.x 23163348 PMC3970115

[5] Yong D, Toleman MA, Giske CG, Cho HS, Sundman K, Lee K, et al Characterization of a New Metallo-beta-Lactamase Gene, bla(NDM-1), and a Novel Erythromycin Esterase Gene Carried on a Unique Genetic Structure in Klebsiella pneumoniae Sequence Type 14 from India. Antimicrobial Agents and Chemotherapy. 2009;53(12):5046–54. 10.1128/AAC.00774-09 19770275 PMC2786356

[6] Walsh TR, Toleman MA, Poirel L, Nordmann P. Metallo-β-Lactamases: the Quiet before the Storm? Clinical Microbiology Reviews. 2005;18(2):306–25. 10.1128/CMR.18.2.306-325.2005 15831827 PMC1082798

[7] Rolain JM, Parola P, Cornaglia G. New Delhi metallo-beta-lactamase (NDM-1): towards a new pandemia? Clinical Microbiology and Infection. 2010;16(12):1699–701. 10.1111/j.1469-0691.2010.03385.x 20874758

[8] Cornaglia G, Giamarellou H, Rossolini GM. Metallo-β-lactamases: a last frontier for β-lactams? The Lancet Infectious Diseases. 2011;11(5):381–93. 10.1016/S1473-3099(11)70056-1 21530894

[9] Mojica MF, Bonomo RA, Fast W. B1-Metallo-beta-Lactamases: Where Do We Stand? Current Drug Targets. 2016;17(9):1029–50. 10.2174/1389450116666151001105622 26424398 PMC4814356

[10] Makena A, Duzgun AO, Brem J, McDonough MA, Rydzik AM, Abboud MI, et al Comparison of Verona Integron-Borne Metallo-beta-Lactamase (VIM) Variants Reveals Differences in Stability and Inhibition Profiles. Antimicrobial Agents and Chemotherapy. 2016;60(3):1377–84. 10.1128/aac.01768-15 26666919 PMC4775916

[11] Verbist L. In vitro activity of temocillin (BRL 17421), a novel beta-lactamase-stable penicillin. Antimicrobial Agents and Chemotherapy. 1982;22(1):157 10.1128/AAC.22.1.157 6982023 PMC183693

[12] Drawz SM, Bonomo RA. Three Decades of beta-Lactamase Inhibitors. Clinical Microbiology Reviews. 2010;23(1):160–+. 10.1128/CMR.00037-09 20065329 PMC2806661

[13] Meini M-R, Llarrull LI, Vila AJ. Overcoming differences: The catalytic mechanism of metallo-β-lactamases. FEBS letters. 2015;589(22):3419–32. Epub 2015/08/20. 10.1016/j.febslet.2015.08.015 . 26297824 PMC4640939

[14] Kim SK, Sims CL, Wozniak SE, Drude SH, Whitson D, Shaw RW. Antibiotic Resistance in Bacteria: Novel Metalloenzyme Inhibitors. Chemical Biology & Drug Design. 2009;74(4):343–8. 10.1111/j.1747-0285.2009.00879.x 19751419

[15] Wang Z, Fast W, Valentine AM, Benkovic SJ. Metallo-β-lactamase: structure and mechanism. Current Opinion in Chemical Biology. 1999;3(5):614–22. 10.1016/S1367-5931(99)00017-4. 10508665

[16] King AM, Reid-Yu SA, Wang W, King DT, De Pascale G, Strynadka NC, et al Aspergillomarasmine A overcomes metallo-beta-lactamase antibiotic resistance. Nature. 2014;510(7506):503–+. 10.1038/nature13445 24965651 PMC4981499

[17] Hinchliffe P, Gonzalez MM, Mojica MF, Gonzalez JM, Castillo V, Saiz C, et al Cross-class metallo-beta-lactamase inhibition by bisthiazolidines reveals multiple binding modes. Proceedings of the National Academy of Sciences of the United States of America. 2016;113(26):E3745–E54. 10.1073/pnas.1601368113 27303030 PMC4932952

[18] Brem J, van Berkel SS, Aik W, Rydzik AM, Avison MB, Pettinati I, et al Rhodanine hydrolysis leads to potent thioenolate mediated metallo-β-lactamase inhibition. Nature chemistry. 2014;6(12):1084–90. 10.1038/nchem.2110 . 25411887

[19] Lahiri S, Panja A, Dasgupta D. Association of a Zn2+ containing metallo beta-lactamase with the anticancer antibiotic mithramycin. Journal of Inorganic Biochemistry. 2015;142:75–83. 10.1016/j.jinorgbio.2014.10.001 25450021

[20] Sohier Jean S, Laurent C, Chevigné A, Pardon E, Srinivasan V, Wernery U, et al Allosteric inhibition of VIM metallo-β-lactamases by a camelid nanobody. Biochemical Journal. 2013;450(3):477 10.1042/BJ20121305 23289540

[21] Kruspe S, Mittelberger F, Szameit K, Hahn U. Aptamers as Drug Delivery Vehicles. Chemmedchem. 2014;9(9):1998–2011. 10.1002/cmdc.201402163 25130604

[22] Zhou JH, Rossi J. Aptamers as targeted therapeutics: current potential and challenges (vol 16, pg 181, 2017). Nature Reviews Drug Discovery. 2017;16(6):440-. 10.1038/nrd.2017.86 28450742

[23] Breaker RR. DNA aptamers and DNA enzymes. Current Opinion in Chemical Biology. 1997;1(1):26–31. 10.1016/S1367-5931(97)80105-6. 9667831

[24] Lakhin AV, Tarantul VZ, Gening LV. Aptamers: Problems, Solutions and Prospects. Acta Naturae. 2013;5(4):34–43. 24455181 PMC3890987

[25] Tuerk C, Gold L. Systematic evolution of ligands by exponential enrichment: RNA ligands to bacteriophage T4 DNApolymerase. Science. 1990;249(4968):505–10. 2200121 10.1126/science.2200121

[26] Davies RB, Abraham EP, Fleming J, Pollock MR. Comparison of beta-lactamase-II from Bacillus cereus 569-H-9 with a beta-lactamase from Bacillus-cereus 5-B-6. Biochemical Journal. 1975;145(2):409–11. 10.1042/bj1450409 808215 PMC1165233

[27] Sabath LD, Abraham EP. Zinc as a cofactor for cephalosporinase from Bacillus cereus 569. Biochemical Journal. 1966;98(1):C11–&. 10.1042/bj0980011C PMC1264844 4957174

[28] Carfi A, Pares S, Duée E, Galleni M, Duez C, Frère JM, et al The 3-D structure of a zinc metallo-beta-lactamase from Bacillus cereus reveals a new type of protein fold. The EMBO journal. 1995;14(20):4914–21. . 7588620 10.1002/j.1460-2075.1995.tb00174.x PMC394593

[29] Fabiane SM, Sohi MK, Wan T, Payne DJ, Bateson JH, Mitchell T, et al Crystal Structure of the Zinc-Dependent β-Lactamase from Bacillus cereus at 1.9 Å Resolution: Binuclear Active Site with Features of a Mononuclear Enzyme. Biochemistry. 1998;37(36):12404–11. 10.1021/bi980506i 9730812

[30] Davies RB, Abraham EP, Melling J. Separation, purification and properties of β-lactamase I and β-lactamase II from Bacillus cereus 569/H/9. Biochemical Journal. 1974;143(1):115–27. 4219278 10.1042/bj1430115 PMC1168359

[31] Lowry OH, Rosebrough NJ, Farr AL, and, Randall RJ. Protein measurement with the Folin phenol reagent. Journal of Biological Chemistry. 1951;193(1):265–75. 14907713

[32] Azatian SB, Kaur N, Latham MP. Increasing the buffering capacity of minimal media leads to higher protein yield. Journal of Biomolecular NMR. 2019 10.1007/s10858-018-00222-4 30613903 PMC6441617

[33] Swift ML. GraphPad prism, data analysis, and scientific graphing. Journal of Chemical Information and Computer Sciences. 1997;37(2):411–2. 10.1021/ci960402j

[34] Kabsch W. Evaluation of single-crystal X-ray-diffraction data from a position-sensitive detector. Journal of Applied Crystallography. 1988;21:916–24. 10.1107/s0021889888007903

[35] Evans PR, Murshudov GN. How good are my data and what is the resolution? Acta Crystallographica Section D-Biological Crystallography. 2013;69:1204–14. 10.1107/s0907444913000061 23793146 PMC3689523

[36] Adams PD, Afonine PV, Bunkoczi G, Chen VB, Davis IW, Echols N, et al PHENIX: a comprehensive Python-based system for macromolecular structure solution. Acta Crystallographica Section D-Biological Crystallography. 2010;66:213–21. 10.1107/s0907444909052925 20124702 PMC2815670

[37] Emsley P, Lohkamp B, Scott WG, Cowtan K. Features and development of Coot. Acta Crystallographica Section D-Biological Crystallography. 2010;66:486–501. 10.1107/s0907444910007493 20383002 PMC2852313

[38] Kay LE, Ikura M, Tschudin R, Bax A. Three-dimensional triple-resonance NMR spectroscopy of isotopically enriched proteins. Journal of Magnetic Resonance (1969). 1990;89(3):496–514. 10.1016/0022-2364(90)90333-5. 22152361

[39] Grzesiek S, Bax A. An efficient experiment for sequential backbone assignment of medium-sized isotopically enriched proteins. Journal of Magnetic Resonance. 1992;99(1):201–7. 10.1016/0022-2364(92)90169-8

[40] Delaglio F, Grzesiek S, Vuister GW, Zhu G, Pfeifer J, Bax A. NMRPIPE—A multidimensional spectral processing system based on unix pipes. Journal of Biomolecular Nmr. 1995;6(3):277–93. 10.1007/bf00197809 8520220

[41] Vranken WF, Boucher W, Stevens TJ, Fogh RH, Pajon A, Llinas P, et al The CCPN data model for NMR spectroscopy: Development of a software pipeline. Proteins-Structure Function and Bioinformatics. 2005;59(4):687–96. 10.1002/prot.20449 15815974

[42] Shen Y, Delaglio F, Cornilescu G, Bax A. TALOS+: A hybrid method for predicting protein backbone torsion angles from NMR chemical shifts. Journal of biomolecular NMR. 2009;44(4):213–23. 10.1007/s10858-009-9333-z 19548092 PMC2726990

[43] DeLano WL. PyMOL: An Open-Source Molecular Graphics Tool2002. 82–92 p.

[44] Lange OF, Rossi P, Sgourakis NG, Song YF, Lee HW, Aramini JM, et al Determination of solution structures of proteins up to 40 kDa using CS-Rosetta with sparse NMR data from deuterated samples. Proceedings of the National Academy of Sciences of the United States of America. 2012;109(27):10873–8. 10.1073/pnas.1203013109 22733734 PMC3390869

[45] Palmer AG, Cavanagh J, Wright PE, Rance M. Sensitivity improvement in proton-detected two-dimensional heteronuclear correlation NMR spectroscopy. Journal of Magnetic Resonance (1969). 1991;93(1):151–70. 10.1016/0022-2364(91)90036-S.

[46] Kay L, Keifer P, Saarinen T. Pure absorption gradient enhanced heteronuclear single quantum correlation spectroscopy with improved sensitivity. Journal of the American Chemical Society. 1992;114(26):10663–5. 10.1021/ja00052a088

[47] Dominguez C, Boelens R, Bonvin A. HADDOCK: A protein-protein docking approach based on biochemical or biophysical information. Journal of the American Chemical Society. 2003;125(7):1731–7. 10.1021/ja026939x 12580598

[48] Patro LPP, Kumar A, Kolimi N, Rathinavelan T. 3D-NuS: A Web Server for Automated Modeling and Visualization of Non-Canonical 3-Dimensional Nucleic Acid Structures. Journal of Molecular Biology. 2017;429(16):2438–48. 10.1016/j.jmb.2017.06.013 28652006

[49] Galleni M, Lamotte-Brasseur J, Rossolini GM, Spencer J, Dideberg O, Frère J-M, et al Standard Numbering Scheme for Class B β-Lactamases. Antimicrobial Agents and Chemotherapy. 2001;45(3):660–3. 10.1128/AAC.45.3.660-663.2001 11181339 PMC90352

[50] Garau G, Garcia-Saez I, Bebrone C, Anne C, Mercuri P, Galleni M, et al Update of the standard numbering scheme for class B beta-lactamases. Antimicrobial Agents and Chemotherapy. 2004;48(7):2347–9. 10.1128/AAC.48.7.2347-2349.2004 15215079 PMC434215

[51] Shaw RW, Clark SD, Hilliard NP, Harman JG. Hyperexpression in Escherichia coli, purification, and characterization of the metallo-β-lactamase of Bacillus cereus 5/B/6. Protein Expression and Purification. 1991;2(2):151–7. 10.1016/1046-5928(91)90064-P. 1821784

[52] Dougall IG, Unitt J. Chapter 2—Evaluation of the Biological Activity of Compounds: Techniques and Mechanism of Action Studies A2—Wermuth, Camille Georges In: Aldous D, Raboisson P, Rognan D, editors. The Practice of Medicinal Chemistry (Fourth Edition). San Diego: Academic Press; 2015 p. 15–43.

[53] Sperka T, Pitlik J, Bagossi P, Tozser J. Beta-lactam compounds as apparently uncompetitive inhibitors of HIV-1 protease. Bioorganic & Medicinal Chemistry Letters. 2005;15(12):3086–90. 10.1016/j.bmcl.2005.04.020 15893929

[54] Widmann M, Pleiss J, and Oelschlaeger P. Systematic Analysis of Metallo-β-Lactamases Using an Automated Database. Antimicrobial Agents and Chemotherapy. 2012;56(7):3481–91. 10.1128/AAC.00255-12 22547615 PMC3393435

[55] Lisi GP, Loria JP. Solution NMR Spectroscopy for the Study of Enzyme Allostery. Chemical Reviews. 2016;116(11):6323–69. 10.1021/acs.chemrev.5b00541 26734986 PMC4937494

[56] Lisa MN, Palacios AR, Aitha M, Gonzalez MM, Moreno DM, Crowder MW, et al A general reaction mechanism for carbapenem hydrolysis by mononuclear and binuclear metallo-beta-lactamases. Nature Communications. 2017;8 10.1038/s41467-017-00601-9 28912448 PMC5599593

[57] Feng H, Liu X, Wang S, Fleming J, Wang D-C, Liu W. The mechanism of NDM-1-catalyzed carbapenem hydrolysis is distinct from that of penicillin or cephalosporin hydrolysis. Nature Communications. 2017;8(1):2242 10.1038/s41467-017-02339-w 29269938 PMC5740130

[58] Mercuri PS, Bouillenne F, Boschi L, Lamotte-Brasseur J, Amicosante G, Devreese B, et al Biochemical Characterization of the FEZ-1 Metallo-β-Lactamase of Legionella gormanii ATCC 33297(T) Produced in Escherichia coli. Antimicrobial Agents and Chemotherapy. 2001;45(4):1254–62. 10.1128/AAC.45.4.1254-1262.2001 11257043 PMC90452

[59] Feng H, Ding JJ, Zhu DY, Liu XH, Xu XY, Zhang Y, et al Structural and Mechanistic Insights into NDM-1 Catalyzed Hydrolysis of Cephalosporins. Journal of the American Chemical Society. 2014;136(42):14694–7. 10.1021/ja508388e 25268575

[60] Tomatis PE, Rasia RM, Segovia L, Vila AJ. Mimicking natural evolution in metallo-beta-lactamases through second-shell ligand mutations. Proceedings of the National Academy of Sciences of the United States of America. 2005;102(39):13761–6. 10.1073/pnas.0503495102 16172409 PMC1236536

[61] Oelschlaeger P, Schmid RD, Pleiss J. Modeling domino effects in enzymes: Molecular basis of the substrate specificity of the bacterial metallo-beta-lactamases IMP-1 and IMP-6. Biochemistry. 2003;42(30):8945–56. 10.1021/bi0300332 12885227

[62] Gonzalez MM, Abriata LA, Tomatis PE, Vila AJ. Optimization of Conformational Dynamics in an Epistatic Evolutionary Trajectory. Molecular Biology and Evolution. 2016;33(7):1768–76. 10.1093/molbev/msw052 26983555 PMC5854100

[63] Sun HG, Zhu X, Lu PY, Rosato RR, Tan W, Zu YL. Oligonucleotide Aptamers: New Tools for Targeted Cancer Therapy. Molecular Therapy-Nucleic Acids. 2014;3 10.1038/mtna.2014.32 25093706 PMC4221593

[64] Fichou Y, Ferec C. The potential of oligonucleotides for therapeutic applications. Trends in Biotechnology. 2006;24(12):563–70. 10.1016/j.tibtech.2006.10.003 17045686

[65] Choi SJ, Ban C. Crystal structure of a DNA aptamer bound to PvLDH elucidates novel single-stranded DNA structural elements for folding and recognition. Scientific Reports. 2016;6 10.1038/srep34998 27725738 PMC5057103

[66] Gitlin I, Carbeck JD, Whitesides GM. Why Are Proteins Charged? Networks of Charge–Charge Interactions in Proteins Measured by Charge Ladders and Capillary Electrophoresis. Angewandte Chemie International Edition. 2006;45(19):3022–60. 10.1002/anie.200502530 16619322

[67] Sharma N, Hu Z, Crowder MW, Bennett B. Conformational Changes in the Metallo-β-lactamase ImiS During the Catalytic Reaction: An EPR Spectrokinetic Study of Co(II)-Spin Label Interactions. Journal of the American Chemical Society. 2008;130(26):8215–22. 10.1021/ja0774562 18528987 PMC2574873

[68] Rydzik AM, Brem J, van Berkel SS, Pfeffer I, Makena A, Claridge TDW, et al Monitoring Conformational Changes in the NDM-1 Metallo-β-lactamase by (19)F NMR Spectroscopy. Angewandte Chemie (International Ed in English). 2014;53(12):3129–33. 10.1002/anie.201310866 24615874 PMC4499255

[69] Abdel-Magid AF. Allosteric Modulators: An Emerging Concept in Drug Discovery. ACS Medicinal Chemistry Letters. 2015;6(2):104–7. 10.1021/ml5005365 25699154 PMC4329591

[70] King JR, Zha JH, Khatri A, Dutta S, Menon RM. Clinical Pharmacokinetics of Dasabuvir. Clinical Pharmacokinetics. 2017;56(10):1115–24. 10.1007/s40262-017-0519-3 28258380

[71] de Bethune MP. Non-nucleoside reverse transcriptase inhibitors (NNRTIs), their discovery, development, and use in the treatment of HIV-1 infection: A review of the last 20 years (1989–2009). Antiviral Research. 2010;85(1):75–90. 10.1016/j.antiviral.2009.09.008 19781578

[72] Karsisiotis AI, Damblon CF, Roberts GCK. Solution structures of the Bacillus cereus metallo-beta-lactamase BcII and its complex with the broad spectrum inhibitor R-thiomandelic acid. Biochemical Journal. 2013;456:397–407. 10.1042/BJ20131003 24059435 PMC3898119

[73] Kim S-K, Demuth M, Schlesinger SR, Kim SJ, Urbanczyk J, Shaw RW, et al Inhibition of Bacillus anthracis metallo-β-lactamase by compounds with hydroxamic acid functionality. Journal of Enzyme Inhibition and Medicinal Chemistry. 2016:1–6. 10.1080/14756366.2016.1222580 27557855

[74] Grover AK. Use of Allosteric Targets in the Discovery of Safer Drugs. Medical Principles and Practice. 2013;22(5):418–26. 10.1159/000350417 23711993 PMC5586781

